# Emerging Roles of m^6^A RNA Methylation Regulators in Gynecological Cancer

**DOI:** 10.3389/fonc.2022.827956

**Published:** 2022-01-28

**Authors:** Wanjun Huang, Fanhua Kong, Ruolan Li, Xiang Chen, Kunpeng Wang

**Affiliations:** ^1^Department of Obstetrics and Gynecology, Taizhou Central Hospital (Taizhou University, Hospital), Taizhou, China; ^2^Zhongnan Hospital of Wuhan University, Institute of Hepatobiliary Diseases of Wuhan University, Transplant Center of Wuhan University, National Quality Control Center for Donated Organ Procurement, Hubei Key Laboratory of Medical Technology on Transplantation, Hubei Clinical Research Center for Natural Polymer Biological Liver, Hubei Engineering Center of Natural Polymer-based Medical Materials, Wuhan, China; ^3^Department of Anesthesiology, Xiangya Hospital of Central South University, Changsha, China; ^4^National Clinical Research Center for Geriatric Disorders, Xiangya Hospital, Changsha, China; ^5^Department of General Surgery, Taizhou Central Hospital (Taizhou University Hospital), Taizhou, China

**Keywords:** gynecological cancer, N^6^-methyladenosine (m^6^A), epigenetics, cervical cancer, endometrial cancer, ovarian cancer

## Abstract

Gynecological cancers seriously affect the reproductive system of females; diseases include ovarian tumors, uterine tumors, endometrial cancers, cervical cancers, and vulva and vaginal tumors. At present, the diagnosis methods of gynecological cancer are insufficiently sensitive and specific, leading to failure of early disease detection. N^6^-methyladenosine (m^6^A) plays various biological functions in RNA modification and is currently studied extensively. m^6^A modification controls the fate of transcripts and regulates RNA metabolism and biological processes through the interaction of m^6^A methyltransferase (“writer”) and demethylase (“erasers”) and the binding protein decoding m^6^A methylation (“readers”). In the field of epigenetics, m^6^A modification is a dynamic process of reversible regulation of target RNA through its regulatory factors. It plays an important role in many diseases, especially cancer. However, its role in gynecologic cancers has not been fully investigated. Thus, we review the regulatory mechanism, biological functions, and therapeutic prospects of m^6^A RNA methylation regulators in gynecological cancers.

## 1 Introduction

Gynecological cancers are a series of tumors that seriously damage the female reproductive system; diseases include ovarian cancer, uterine cancer, endometrial cancer, cervical cancer, and vulva and vaginal cancer (1). Gynecological cancers become a serious global public health challenge due to their high incidence in women of all ages (2). Among them, ovarian cancer, endometrial cancer, and cervical cancer are the most common gynecological tumors. Considerable studies have shown that the occurrence and development of gynecological cancers are related to the activation of oncogenes, the inactivation of tumor suppressor genes, and the activation of abnormal cell signaling pathways. In addition, epigenetic processes regulate gene expression through DNA methylation, histone modification, and noncoding RNA, thereby affecting the occurrence and development of gynecological cancer (3).

Ovarian cancer is one of the most common malignant tumors in women (3). It has the characteristic of insidious onset and has no specific clinical symptoms in the early stage of the disease. Moreover, sensitive and effective clinical screening methods for ovarian cancer are currently limited, and approximately 70% of patients are advanced at the time of diagnosis (4). According to the American Cancer Society, the United States records approximately 21,000 new cases of ovarian Cancer each year, accounting for 5% of all female malignancies, with a mortality rate of 62% and a five-year survival rate of 20%–30%, seriously affecting women’s health (5). At present, the pathogenesis of ovarian cancer is still unclear. The development of epigenetics provides new means to discover specific biomarkers and treatment methods, which greatly improve the diagnosis and treatment prospects of ovarian cancer.

Cervical cancer is the fourth most common female malignancy in the world and a leading cause of cancer-related death in women (6, 7). Cervical cancer is diagnosed in more than 500,000 patients worldwide each year and leads to more than 300,000 deaths (8, 9). Although HPV vaccination is effective in preventing cervical cancer, it remains the fourth most common cancer among women globally due to inadequate screening programs in many parts of the world (10, 11). Despite the continuous innovation of radiotherapy and/or chemotherapy on the basis of surgery, early lymph node metastasis still occurs in some patients with cervical cancer, resulting in poor prognosis and low survival rate. The five-year survival rate is still approximately 40%, posing a serious threat to women’s health (12–15). Thus, elucidating the molecular mechanism of cervical cancer occurrence and metastasis has great clinical importance.

Endometrial cancer, as one of the most common gynecological cancers, has become the fourth most common malignant tumor and the fifth most common cause of death among women in the United States (16, 17). According to WHO statistics in 2021, the total incidence of endometrial cancer in the United States was 7%, with 66,570 new cases (5). Despite advances in drugs and surgical treatments for endometrial cancer, recent studies have shown that survival rates for endometrial cancer have not improved significantly, but death rates have increased. Thus, improving the ability to identify the prognostic risk factors of endometrial cancer and formulating reasonable new treatment plans are greatly important for improving the survival rate and prognosis of patients with endometrial cancer (18). In recent years, the study of tumor genesis, intracellular signaling pathway changes, and epigenetics in tumor microenvironment has developed rapidly, providing a new means for the discovery of specific biomarkers and therapeutic methods (19).

m^6^A methylation was first identified in 1974 and subsequently proven to be the most common and abundant RNA modification in eukaryotic cells (20). m^6^A modification not only exists in mRNA, but also in various noncoding RNAs (21, 22). m^6^A modification affects cell function by regulating the function and metabolism of RNA; it is involved in various pathophysiological processes, such as cell division, immune regulation, and regulation of the occurrence of various cancers (23, 24). A large number of studies have shown that m^6^A modification is related to the proliferation, differentiation, tumorigenesis, invasion, and metastasis of gynecological cancers; it can function as an oncogene or anticancer gene (25–28). Here, we comprehensively review the modification of m^6^A and analyze the potential molecular mechanism of m^6^A in gynecological cancers. The prospect of m^6^A modification as a new marker and therapeutic target for gynecological cancer was further clarified.

## 2 Molecular Mechanisms of m^6^A Modification

### 2.1 m^6^A Writers

**Figure 1:**
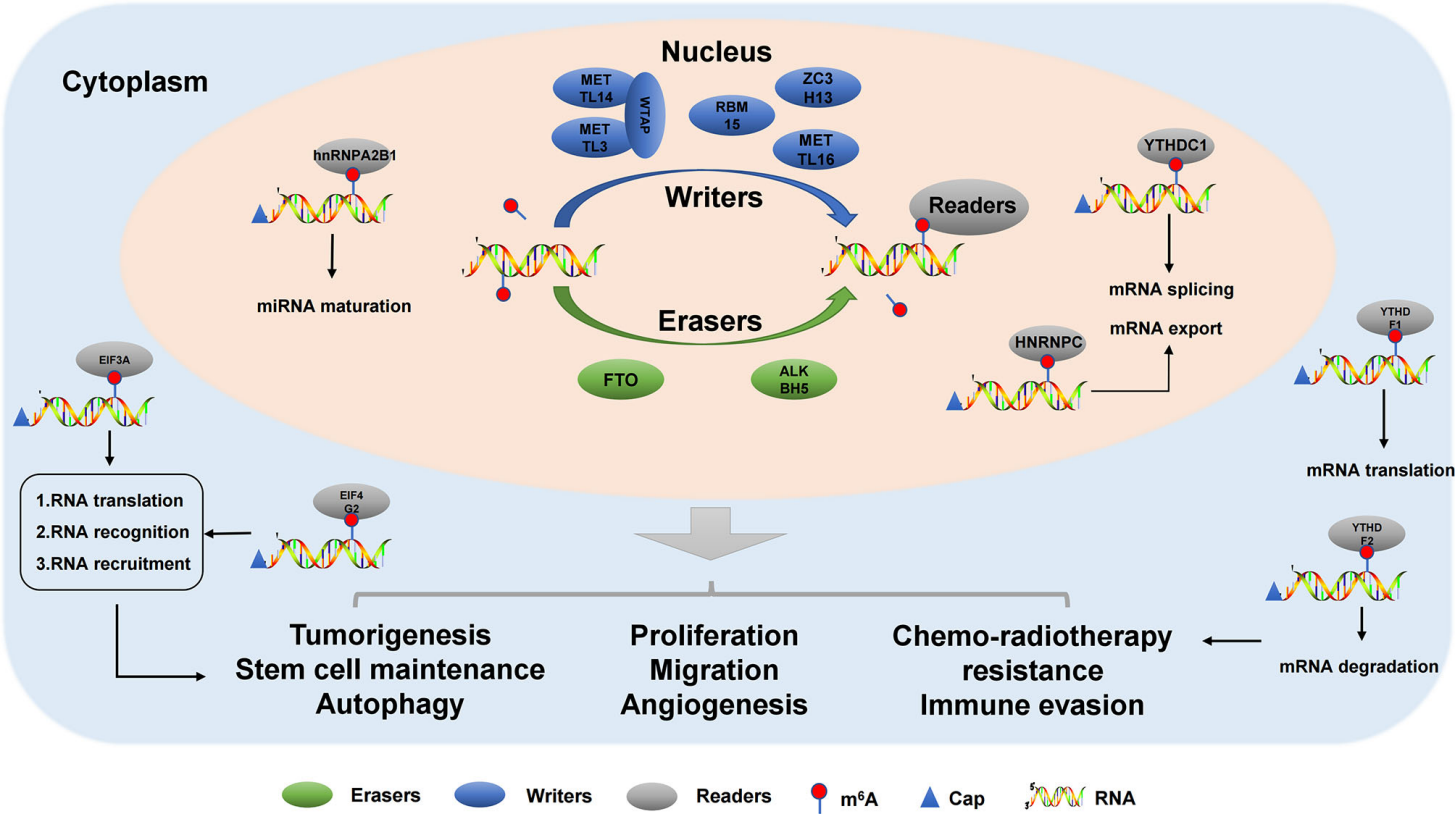
The molecular mechanisms of m^6^A modification in cancers. m^6^A modification is a dynamic and reversible process. m^6^A methylation is catalysed by methyltransferase complex (writers), reversed by demethylases (erasers) and functionally facilitated by m^6^A-binding proteins (readers). m^6^A methylation participates in carcinogenesis and tumor progression.

Modification of m^6^A is dynamic and reversible, and methyltransferases (writers) are mainly composed of KIAA1429(VIRMA), METTL3, RBM15, WTAP, ZC3H13, METTL16, METTL14, and CBLL1 (29). KIAA1429, also known as Virlike m^6^A methyltransferase-associated protein (VIRMA), a newly identified component of the RNA m^6^A methyltransferase complex, plays a key role in guiding regionally selective m^6^A deposition (30). Meanwhile, it regulates the expression of sex-lethal genes by selective splicing of premRNA with WTAP (31). Interestingly, METTL14 and METTL3, as core components of m^6^A methyltransferase, form A stable METTL3-METTL14 heterodimer core complex, which plays A role in cell m^6^A deposition on mammalian nuclear RNA (32). As a mammalian splicing factor, WTAP has no methylation activity by itself, but can interact with METTL14/METTL3 complex and affect methylation (32). In addition, WTAP is a regulatory subunit required for the formation of the m^6^A methyltransferase complex (including METTL3 and METTL14), which plays an important role in gene expression regulation and alternative splicing. Moreover, *in vivo* localization to pre-mRNA rich nuclear spots and catalytic m^6^A methyl transferase activity. In the absence of WTAP, the RNA binding ability of METTL3 was significantly weakened (33).The main feature of METTL3 and METTL14 is that they contain methyltransferase domains (S-adenosine methylene thiocyanine binding motifs, SAM-binding), which transfer methyl groups to adenosine at N^6^ (34, 35). RBM15, an RNA-binding protein, is involved in m^6^A modification and the regulation of alternative splicing (AS) through the regulation of Notch, Wnt, and other signaling pathways; it has inhibitory functions in multiple signaling pathways (36). ZC3H13 is a typical CCCH zinc finger protein, which acts as a tumor suppressor and inhibits tumor development by regulating Ras-ERK signaling pathway (37). METTTL16 is an emerging player in the field of RNA modification in human cells. Originally thought to be a ribosomal RNA methyltransferase, it has currently been shown to bind and methylate MAT2A messenger RNA (mRNA) and U6 small nuclear RNA (snRNA) (38). Casitas B family lymphoma transforming sequence-like protein 1 (CBLL1), also known as Hakai, was originally identified as the E3 ubiquitin ligase of the E-cadherin complex (39). The molecular mechanisms of m^6^A writers as show in **Figure 1**.

### 2.2 m^6^A Erasers

Thus far, only two m^6^A demethylases have been identified, namely, ALKBH5 and FTO (40). ALKHB5 is a member of the AlkB family and plays an important regulatory role in many biological processes, such as mRNA modification and regulation (41, 42). ALKBH5 also plays a regulatory role in the occurrence and development of tumors (41). For example, ALKBH5 inhibits pancreatic cancer by decreasing WIF-1 RNA methylation and mediating Wnt signaling (43). ALKBH5 promotes the invasion and metastasis of gastric cancer by reducing the methylation of lncRNA NEAT1 (44). At the same time, autophagy in epithelial ovarian cancer was inhibited by miR-7 and Bcl-2 (45). In addition, ALKBH5 can regulate the expression of PD-L1 in cholangiocarcinoma, promote the expression of PD-L1 on monocytes/macrophages, and reduce the infiltration of bone marrow-derived inhibitory cells, making tumors with strong ALKBH5 nuclear expression pattern more sensitive to PD1 immunotherapy (46). Fat mass and obesity-related protein (FTO), as the first m^6^A demethylase responsible for RNA modification in cells, is involved in various physiological processes, and its dysregulation is closely related to various human diseases, especially the occurrence and development of tumors (47). The FTO gene was originally identified as being involved in obesity and type 2 diabetes. This gene encodes the FTO protein, belonging to the AlkB dioxygenase family dependent on Fe^2+^ and 2-oxoglutarate (2OG) (48). FTO shows complex biological functions in physiological process, and its disorder is related to various human diseases (49). The molecular mechanisms of m^6^A erasers as show in **Figure 1**.

### 2.3 m^6^A Readers

m^6^A binding protein, composed of YT521-B homolog (YTH) domain protein, is mainly composed of HNRNP family (HNRNPA2/B1, HNRNPC, and HNRNPG) and YTH domain protein family (YTHDC1, YTHDC2, YTHDF1, YTHDF2, and YTHDF3). In addition, it includes FXR, IGF2BP, eIF, and G3BP family members (50). Heterogeneous nuclear ribonucleoproteins (hnRNPs) represent a large family of RNA-binding proteins (RBPs) that play roles in nucleic acid metabolism including selective splicing, mRNA stabilization, transcription, and translation regulation (51). The expression levels of hnRNPs are altered in many types of cancer, suggesting that they play an important regulatory role in tumorigenesis. The YTH domain recognizes m^6^A modifications through a conserved aromatic ring. This RNA binding domain is dependent on m^6^A modification (52). Reading proteins recognize and read information from m^6^A RNA in a methylation-dependent manner. YTHDC1-2 and YTHDF1-3 are the main intracellular proteins in the human body. YTHDC2 is located in the cytoplasm of meiosis spermatocytes, and YTHDF1-3 mainly recognizes the information of m^6^A methylation in the cytoplasm (53, 54). The five proteins not only contain the same domain, but also have special domains that determine their different roles. The molecular mechanisms of m^6^A readers as show in **Figure 1**.

## 3 m^6^A and Cancers

Previous studies have shown that the role of m^6^A methylation regulators in carcinogenesis and tumor progression is mainly achieved by regulating oncogene expression and inhibiting gene expression (40). m^6^A methylation regulator plays a “double-edged sword” role in tumor progression, which can promote the expression of oncogenes and inhibit the expression of oncogenes to promote tumor progression or the expression of oncogenes and inhibit the expression of oncogenes to inhibit tumor progression (55). With the deepening of studies on m^6^A methylation, the pathophysiological processes of m^6^A modification and regulation also expand, including mRNA regulation, immune regulation, biorhythm, neural development, and autophagy (21, 56). The dual role of m^6^A in cancer is increasingly recognized (57, 58). On the one hand, m^6^A regulates the expression of oncogenes or tumor suppressor genes, thereby affecting tumor progression. On the other hand, the level of m^6^A and the expression and activity of m^6^A enzyme can be regulated, affecting the role of m^6^A in cancer (58). Thus, m^6^A methylation regulator is expected to be a potential target for cancer therapy. The relationship between m^6^A modification and various gynecological cancers is reviewed, and the role of m^6^A regulatory factors in different gynecological cancers is further clarified.

### 3.1 m^6^A in Cervical Cancer

#### 3.1.1 Function of m^6^A on mRNA in Cervical Cancer

Post-expression regulation of genes is mainly carried out in four aspects, namely, transcription, post-transcription, translation, and post-translation. m^6^A modification is mainly manifested in the RNA transcription process, which regulates gene expression after RNA transcription by modifying the structure of RNA or specific binding in the form of binding protein (59). As an important m^6^A “reader”, YTHDF1 regulates the fate of m^6^A-modified mRNA. Studies have found that the up-regulation of YTHDF1 in cervical cancer is closely related to the poor prognosis of cervical cancer patients. YTHDF1 regulates RANBP2 translation in an m^6^A-dependent manner without affecting its mRNA expression. RANBP2 can promote the growth, migration, and invasion of cervical cancer cells. Thus, YTHDF1 has a carcinogenic effect in cervical cancer by regulating the expression of RANBP2, and YTHDF1 is a potential target for cervical cancer treatment (26). YTHDF2, as another member of the YTH domain protein family, is also up-regulated in cervical cancer, and the higher expression in cervical cancer indicates shorter survival time. After YTHDF2 knockdown, the proliferation of cervical cancer cells was transplanted to promote cell apoptosis, and the tumor cells stagnated in the S phase (60).

**Figure 2:**
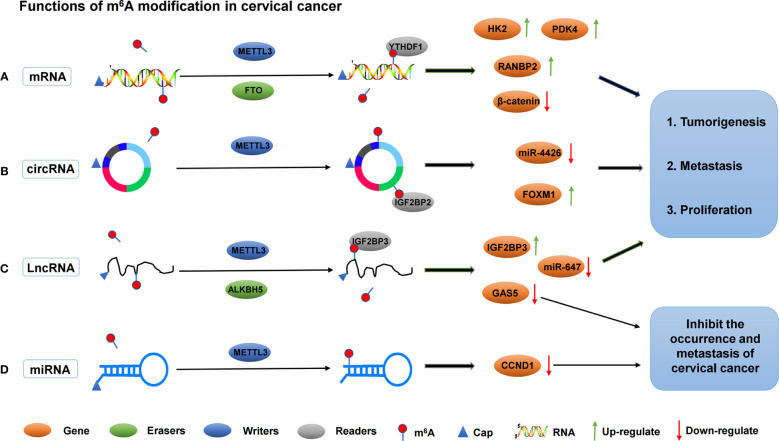
The roles of different m^6^A regulators in regulating RNAs in cervical cancer. **(A)** m^6^A methylation regulators METTL3, FTO and YTHDF1 promote the invasion and metastasis of cervical cancer by binding to mRNA and regulating mRNA expression. **(B)** m^6^A methylation regulators METTL3 and IGF2BP2 promote the invasion and metastasis of cervical cancer by binding to circRNA and regulating target genes expression. **(C)** m^6^A methylation regulators METTL3, ALKBH5 and IGF2BP3 promote/inhibit the invasion and metastasis of cervical cancer by binding to lncRNA and regulating target genes expression. **(D)** m^6^A methylation regulators METTL3 inhibit the invasion and metastasis of cervical cancer by binding to lncRNA and regulating target genes expression.

In addition to m^6^A “readers” that play an important regulatory role in cervical cancer, m^6^A “writers” play an important regulatory role in the occurrence and progression of cervical cancer. For example, METTL3 is significantly up-regulated in cervical cancer tissues and cells; it is closely associated with lymph node metastasis and poor prognosis in cervical cancer patients. Moreover, METTL3 can promote the proliferation and decrease apoptosis of cervical cancer cells *in vitro*. METTL3 promotes the proliferation and aerobic glycolysis of cervical cancer cells by targeting the 3’ -untranslated region (3’-UTR) of hexokinase 2 (HK2) mRNA. In addition, METTL3 in combination with YTHDF1 enhances the stability of HK2. These data suggest that METTTL3 may be a carcinogenic factor in the development of cervical tumors. In the existing studies, the role of METTTL3 in aerobic glycolysis of tumors is rarely reported. Thus, METTTL3 regulates the stability of HK2 mRNA by recruiting YTHDF1 and acts as a carcinogen by accelerating glycolysis through the YTHDF1/HK2 axis, which provides a potential prognostic biomarker for cervical cancer treatment (61). FTO mRNA level, as an m^6^A demethylase, is up-regulated in cervical cancer tissues, and FTO regulates chemotherapy resistance of cervical squamous cell carcinoma (CSCC) through mRNA demethylation targeting β-catenin. FTO regulates β-catenin expression by reducing m^6^A level in β-catenin mRNA transcripts. Furthermore, the excision repair cross-complementary group 1 (ERCC1) activity was improved to enhance chemotherapy and radiotherapy resistance *in vitro* and *in vivo* (62). In addition, Li et al. found that TATA binding protein (TBP) can increase the expression of METTL3 in cervical cancer cells by binding to the promoter of METTL3. *In vivo* and clinical data confirm that m^6^A/PDK4 plays an active role in the growth and progression of cervical cancer and liver cancer. Furthermore, m^6^A regulates glycolysis of cancer cells through PDK4, and the methylation of PDK4 is regulating the stability and translation of its mRNA, thereby regulating glycolysis in cancer cells (63). The roles of different m^6^A regulators in regulating RNAs in cervical cancer are shown in **Figure 2** and **Table 1**.

**Table 1:** The roles of different m^6^A regulators in regulating RNAs in cervical cancer.

m^6^A regulators	Genes/RNAs	Cell lines	Location	Role	Mechanism	Function	References
YTHDF1	RANBP2	HEK293T, Hela, SiHa	mRNA	Oncogene	Enhance expression of RANBP2	Promote cervical cancer tumorigenesis and metastasis	(26)
METTL3	HK2	CaSki, SiHa, C33A, HT-3, HaCaT	mRNA	Oncogene	Enhance expression of HK2	Promote cervical cancer tumorigenesis and aerobic glycolysis	(61)
FTO	β-catenin	SiHa, c-33a	mRNA	Oncogene	down-regulated expression of β-catenin	Enhance the activity of ERCC1	(62)
METTTL3	PDK4	HeLa, SiHa, Huh7, HepG2, MDA-MB-231, ECT1/E6E7	mRNA	Oncogene	TBP promotes the expression of METTL3	Regulating glycolysis in cancer cells	(63)
IGF2BP2	circARHGAP12	HaCaT, HT-3, CaSki, C33A, SiHa	CircRNA	Oncogene	Enhanced the stability of FOXM1 mRNA	Promote cervical cancer tumorigenesis and metastasis	(64)
METTTL3	circ_0000069	SiHa, Caski, C33A, Ect1, 293T	CircRNA	Oncogene	Inhibit the expression of miR-4426	Promote cervical cancer tumorigenesis and metastasis	(65)
ALKBH5	LncRNA GAS5-AS1	Caski, SiHa, C33A, HeLa, HCvEpC	LncRNA	Tumor suppressor	Decreasing GAS5 N6-methyladenosine m(6)A modification	Reduced cervical cancer tumorigenesis and metastasis	(66)
IGF2BP3	KCNMB2-AS1	SiHa, HeLa,	LncRNA	Oncogene	KCNMB2-AS1 competed with miR-130b-5p and miR-4294 and up-regulated IGF2BP3	Promote cervical cancer tumorigenesis and metastasis	(67)
METLL3	ZFAS1	Hela, SiHa, C33A, CaSki, 293T	LncRNA	Oncogene	ZAFS1 sequestered miR-647 and regulated by METLL3	Promote cervical cancer tumorigenesis and metastasis	(68)
METLL3	miR-193b	Siha, Hela	miRNA	Tumor suppressor	By targeting CCND1	Reduced cervical cancer tumorigenesis and metastasis	(69)

#### 3.1.2 Function of m^6^A on ncRNA In Cervical Cancer

Noncoding RNAs (ncRNAs) have been shown to be involved in the development and progression of cervical cancer (65). Circ-RNAs are a class of ncRNAs with covalently closed circular structures, which are generated by reverse splicing of exon precursor mRNA or lasso intron splicing (70, 71). The most studied function of circ-RNA is being a major regulator of gene expression, and its role is to isolate or “sponge” other gene expression regulators, especially miRNAs. They have also been shown to work by directly regulating transcription and interfering with splicing mechanisms (72). At present, with the deepening of people’s understanding of cervical cancer, the pathogenesis of circ-RNA in cervical cancer is widely studied. Fei et al. found, by analyzing cervical cancer RNA sequencing data, that a new m^6^A modified circ-RNA (circARHGAP12, hsa_circ_0000231) was up-regulated in cervical cancer tissues and cells. Further studies found that circARHGAP12 can promote tumor progression of cervical cancer. In addition, circARHGAP12 interacts with m^6^A reader IGF2BP2 to bind to FOXM1 mRNA and enhance the stability of FOXM1 mRNA (64). Another circ-RNA (hsa_circ_0000069) was also regulated by m^6^A modification. Hsa_circ_0000069 expression was up-regulated in cervical cancer, and m^6^A modification enhanced the stability of circ_0000069. The proliferation and migration of cervical cancer cells were promoted by inhibiting miR-4426 (65). miR-4426 expression in cervical cancer cells was down-regulated due to the up-regulation of circ_0000069. Therefore, m^6^A modification indirectly inhibits the expression of miR-4426, which in turn inhibits cell proliferation and migration. However, the downstream targets of miR-4426 remain unclear.

Long ncRNAs (LncRNAs), a group of large transcripts (more than 200 nucleotides in length) without protein-coding potential, play an important role in various human diseases, including cancer. LncRNAs are involved in various pathophysiological processes, such as cell proliferation, migration, invasion, apoptosis, and chemotherapy resistance (73). m^6^A modification is the most abundant internal modification of RNA and exists in various RNAs, such as mRNA and lncRNA. At present, only few lncRNAs have been functionally verified in cervical cancer, especially those regulated by m^6^A modification. For example, the expression of lncRNA GAS5-AS1 is significantly down-regulated in cervical cancer tissues, and studies have found that the down-regulation of GAS5-AS1 is significantly correlated with late, distant, lymphatic metastasis, and poor prognosis of FIGO in cervical cancer patients. Moreover, GAS5-AS1 interacts with ALKBH5, which reduces the m^6^A modification of GAS5 and increases its stability, revealing the important mechanism of epigenetic changes in the occurrence and metastasis of cervical cancer (66). Another lncRNA, KCNMB2-AS1, is significantly overexpressed in cervical cancer and is associated with poor prognosis. KCNMB2-AS1 is predominantly located in the cytoplasm, and leads to upregulation of IGF2BP3, which is a proven oncogene in cervical cancer, as endogenous RNA competes with a large number of miR-130b-5p and miR-4294. In addition, IGF2BP3 binds to KCNMB2-AS1 *via* three m^6^A modification sites on KCNMB2-AS1. IGF2BP3, as the “reader” of m^6^A, plays a stabilizing role in KCNMB2-AS1 and promotes the occurrence and development of cervical cancer (67). LncRNA ZFAS1 has been observed to be abnormally expressed in cervical carcinoma (74). Yang et al. found that the expression of ZFAS1 was up-regulated in cervical cancer, and the up-regulation of ZFAS1 was correlated with FIGO stage, lymph node, and distant metastasis. This finding also suggested that the overall survival of cervical cancer patients was poor (68). In addition, ZAFS1 isolated miR-647, an RNA–RNA interaction regulated by METTL3-mediated m^6^A modification. Another study found that METTL3 enhanced the stability of lncRNA FOXD2-AS1 and maintained its expression, and promoted the development of cervical cancer. FOXD2-AS1 is significantly up-regulated in cervical cancer cells and tissues, which is closely associated with poor prognosis. Moreover, FOXD2-AS1 promotes the migration and proliferation of cervical cancer cells. Mechanistically, METTL3 enhances the stability of FOXD2-AS1 and maintains its expression. Meanwhile, FOXD2-AS1 recruits LSD1 to be silenced on the promoter of P21, thus accelerating the progression of cervical cancer (75).

MiRNAs are small ncRNAs (18-24 nucleotides) that negatively regulate gene expression by binding to target mRNA in the 3 ‘-untranslated region (UTR) during the post-transcriptional stage. Many physiological and pathophysiological processes, including cancer, are affected by miRNA activity (76). Huang et al. found from the cervical cancer specimens that the low expression of miR-193b was closely related to cervical cancer staging and interstitial invasion. miR-193b, as a tumor suppressor, is regulated by the m^6^A methylation regulator METTL3 in cervical cancer. miR-193b inhibits the occurrence and development of cervical cancer by targeting CCND1 (69). Currently, studies on how m^6^A regulates miRNA expression in cervical cancer are relatively few, and more studies are still required to further reveal the regulatory mechanism of m^6^A in miRNA.

#### 3.1.3 Prognostic Effect of m^6^A RNA Methylation Regulators on Cervical Cancer

Worldwide, cervical cancer remains one of the most common type of cancer with a major treatment challenge facing mankind (77). The carcinogenesis of cervical cancer is a complex multistep process characterized by a wide range of molecular abnormalities, providing many potential therapeutic targets. Understanding the mechanism of these molecules is crucial for their potential therapeutic uses (78, 79). In recent years, RNA modification plays an important role in various biological processes, and its abnormal regulation has become an important factor affecting the occurrence and development of tumors. Several m^6^A RNA methylation regulators have been found to be prognostic factors for various cancers (80, 81). Wu et al. compared the differential expression of 20 m^6^A RNA methylation regulators in cervical cancer tissues by using RNA sequence data and clinical information in TCGA database. Among them, five m^6^A RNA methylation regulators (FTO, HNRNPA2B1, RBM15, IGF2BP1, and IGF2BP3) were significantly correlated with the status of cervical cancer. In addition, six m^6^A RNA methylation regulators (YTHDC2, YTHDC1, ALKBH5, ZC3H13, RBMX, and YTHDF1) were selected to construct risk markers. The overall survival of cervical cancer patients in the high-risk group was significantly lower than that in the low-risk group, with an area under the curve (AUC) of 0.718. Thus, this risk model can be used as an independent prognostic factor for cervical cancer patients; it can predict the overall survival of cervical cancer patients with different clinical factors (82). METTL3 is a member of the m^6^A methyltransferase family, which acts as an oncogene in cancer. Ni et al. found that METTL3 and CD33(+) MDSCs were up-regulated in cervical cancer tissues by analyzing paraffin-embedded tumor specimens from 197 patients with cervical cancer. METTTL3 expression was positively correlated with CD33(+) cell density in tumor tissues. Meanwhile, METTL3 level in tumor microenvironment was significantly correlated with tumor-advanced stage. The levels of METTTL3 and CD33(+) MDSCs in tumor tissue were significantly correlated with the reduction of DFS or OS. Thus, Cox model analysis showed that the METTL3 level in cervical cancer cells was an independent factor for patient survival (83). Pan et al. obtained clinical and survival data and RNA sequencing data of 13 m^6^A RNA methylation regulators from the TCGA database. Consensus cluster analysis was performed to identify different cervical cancer clusters according to the expression differences of regulatory factors. Four regulatory factors (RBM15, METTTL3, FTO, and YTHDF2) were abnormally expressed in cervical cancer tissues. LASSO Cox regression analysis showed that ZC3H13, YTHDC1, and YTHDF1 were independent prognostic indicators of cervical cancer (84).

### 3.2 m^6^A in Endometrial Cancer

#### 3.2.1 Function of m^6^A on mRNA in Endometrial Cancer

m^6^A dynamic methylation mRNA may affect cell physiology, especially key transcripts, which may lead to significant changes in biological functions (49). In recent years, studies have shown that m^6^A modified mRNA methylation plays an important role in cell proliferation and tumorigenicity of endometrial cancer, and the decrease or increase in m^6^A mRNA methylation is likely to be the carcinogenic mechanism of most endometrial cancer, promoting the occurrence and development of endometrial cancer (27). m^6^A-dependent mRNA regulation affects various biological processes in endometrial cancer and is involved in the regulation of RNA structure, translation, and degradation (85). ALKBH5, an RNA demethylase, is significantly up-regulated in endometrial carcinoma and promotes the proliferation and invasion of endometrial carcinoma. Studies have shown that ALKBH5 mainly regulates the demethylation of target gene IGF1R and enhances the stability of IGF1R mRNA, thereby promoting IGF1R translation and activating IGF1R signaling pathway. It further promotes the proliferation and invasion of endometrial cancer, suggesting a potential therapeutic target for endometrial cancer (27). FTO, another demethylase, can eliminate m^6^A modification and regulate the metabolism of mRNA. Although many studies have confirmed the relationship between obesity and endometrial cancer, the molecular mechanism of obesity and endometrial cancer progression has not been clarified. Zhang et al. found that the expression of FTO was up-regulated in endometrial cancer, and this effect promoted the metastasis and invasion of endometrial cancer. In addition, FTO catalyzed the demethylation of the 3’UTR region of HOXB13 mRNA, thereby eliminating the recognition of m6A modification from YTHDF2 protein. Decreased mRNA attenuation of HOXB13 leads to increased protein expression, whereas WNT signaling pathway activation and downstream protein expression lead to metastasis and invasion of endometrial carcinoma (86).

**Figure 3:**
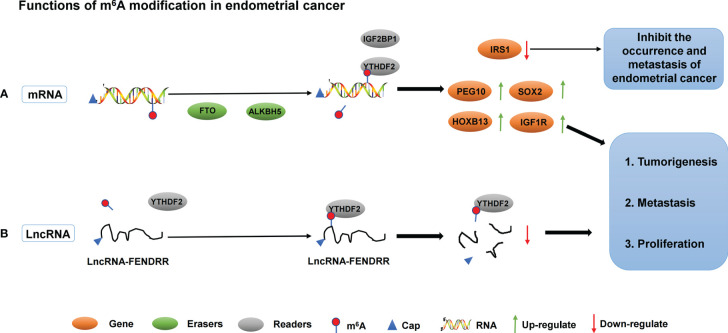
The roles of different m^6^A regulators in regulating RNAs in endometrial cancer. **(A)** m^6^A methylation regulators ALKBH5, FTO, IGF2BP1 and YTHDF2 promote/inhibit the invasion and metastasis of endometrial cancer by binding to mRNA and regulating mRNA expression. **(B)** m^6^A methylation regulators YTHDF2 promote the invasion and metastasis of endometrial cancer by binding to lncRNA-FENDRR and down- regulating lncRNA-FENDRR expression.

In addition to demethylase, methyltransferase plays an important role in the development of endometrial carcinoma. Liu et al. found that approximately 70% of tumor samples from patients with endometrial cancer showed decreased m^6^A levels, either due to reduced METTL3 expression or loss of functional mutations in METTL14. METTL14 mutations and METTL3 downregulation down-regulated m^6^A mRNA methylation levels and enhanced endometrial carcinoma proliferation and tumorigenicity. In addition, downregulation of m^6^A methylation decreases the expression of the negative AKT regulator PHLPP2 and increases the expression of the positive AKT regulator mTORC2, thereby activating the AKT pathway. Therefore, m^6^A modification mediated by METTL14 and METTL3 is a regulator of the AKT signaling pathway (87). In addition, the expression of m^6^A reader protein YTHDF2 was significantly up-regulated in endometrial cancer. YTHDF2 promoted the degradation of IRS1 mRNA by binding to the methylation site of the target transcript of IRS1, thereby inhibiting the expression of IRS1, inhibiting the IRS1/AKT signaling pathway, and ultimately inhibiting the tumorigenicity of endometrial cancer (88). IGF2BP1 belongs to the IGF2BP family. Studies have shown that IGF2BP1 is involved in the regulation of mRNA and affects the function of tumor cells (89). Zhang et al. found that up-regulation of IGF2BP1 expression in endometrial cancer is a factor affecting patient survival. Moreover, IGF2BP1 enhances PEG10 expression and promotes endometrial cancer cell proliferation by recognizing the m^6^A site of PEG10 mRNA (90). Another study found that PADI2-catalyzed MEK1 citrulline activates ERK1/2 and promotes IGF2BP1-mediated SOX2 mRNA stability. PADI2-catalyzed MEK1 R113/189 citrulline is a key factor in endometrial cancer. These findings suggest that targeting PADI2/MEK1 may be a potential therapy for endometrial cancer patients (91). The roles of different m^6^A regulators in regulating RNAs in endometrial cancer are shown in **Figure 3** and **Table 2**.

**Table 2:** The roles of different m^6^A regulators in regulating RNAs in endometrial cancer.

m^6^A regulators	Genes/RNAs	Cell lines	Location	Role	Mechanism	Function	References
ALKBH5	IGF1R	HEC-1-A, RL95-2, T-HESCs	mRNA	Oncogene	Enhance expression of IGF1R	Promote endometrial cancer tumorigenesis and metastasis	(27)
FTO	HOXB13	AN3CA, KLE	mRNA	Oncogene	Enhance expression of HOXB13 and activate the WNT signaling pathway	Promote endometrial cancer tumorigenesis and metastasis	(86)
YTHDF2	IRS1	HEC-1-A, RL95-2, T-HESCs	mRNA	Tumor suppressor	Degrade IRS1 mRNA, Inhibition of IRS1/AKT signaling pathway	Inhibit the tumorigenicity of endometrial carcinoma	(88)
IGF2BP1	PEG10	Ishikawa, HEC-1-A, HEC-1-B, RL-95-2, AN3CA, KLE	mRNA	Oncogene	Enhance expression of PEG10	Promote endometrial cancer tumorigenesis and metastasis	(90)
IGF2BP1	SOX2	Ishikawa, ECC-1, HEK293	mRNA	Oncogene	Enhance expression of SOX2	Promote endometrial cancer tumorigenesis and metastasis	(91)
YTHDF2	FENDRR	Ishikawa, HEC-1-B	LncRNA	Oncogene	Degrade LncRNA FENDRR	Promote endometrial cancer tumorigenesis and metastasis	(92)

#### 3.2.2 Function of m^6^A on ncRNA in Endometrial Cancer

Abnormal regulation of ncRNA has been shown to be closely associated with the progression of endometrial cancer. At present, the study on the relationship between m^6^A and ncRNA in endometrial cancer is still in the early stage. Shen et al. studied 60 cases of endometrial carcinoma from tumor tissues, cell lines, and xenograft mouse models. He found that the LncRNA FENDRR expression level decreased and the m^6^A methylation level increased in the cancer tissues of patients with endometrial cancer. *In vitro* experiments showed that YTHDF2 could recognize the abundance of m^6^A modified LncRNA FENDRR in endometrial cancer cells and promote its degradation. Overexpression of LncRNA FENDRR inhibited the proliferation and promoted apoptosis of HEC-1B cells by reducing the mRNA level of SRY-related HMG box transcription factor 4 (SOX4) protein. *In vivo* experiments confirmed that LncRNA FENDRR overexpression inhibited the growth of endometrial cancer cells. Therefore, in endometrial cancer, the m^6^A modification level of lncRNA FENDRR is increased, and YTHDF2 is recruited to promote the degradation of FENDRR. Subsequently, the downregulation of FENDRR leads to the accumulation of SOX4 protein, thereby promoting the proliferation of EEC cells (92).

#### 3.2.3 Prognostic Effect of m^6^A RNA Methylation Regulators on Endometrial Cancer

Endometrial cancer is the sixth most common cancer in women worldwide. The expression level of m^6^A regulatory factor may be used for stratification of cancer prognosis, including endometrial cancer. Wang et al. determined, by analyzing matched clinical information from the TCGA database of endometrial cancer patients, that replication number variations (CNVs) in the m^6^A regulatory gene had a significant negative impact on patient survival. Univariate Cox regression analysis showed that IGF2BP1, KIAA1429, IGF2BP3, YTHDF3, and IGF2BP2 were closely related to the survival and prognosis of endometrial cancer patients. Among them, IGF2BP3, KIAA1429, and IGF2BP1 can effectively predict the prognosis of patients (93). In addition, Pang et al. found that IGF2BP1 and YTHDF3 had a strong ability to stratify the prognosis of different endometrial cancer patients (94). Song et al. downloaded the human endometrial carcinoma m^6^A sequencing dataset “GSE93911” from the comprehensive gene expression database. A total of 181 genes with significantly differentially expressed and differentially methylated loci in endometrial carcinoma were screened. Among them, 31 genes were associated with survival, and 11 genes were identified as risk prognosis models, including GDF7, BNC2, SLC8A1, B4GALNT3, DHCR24, ESRP1, HOXB9, IGSF9, KIAA1324, MSnX1, and PHGDH (95). In addition, Ma et al. analyzed the sequences, copy number variation, and clinical data obtained from the TCGA database. The changes in the m^6^A RNA methylation regulators are closely related to the clinicopathological stage and prognosis of endometrial carcinoma. Among them, ZC3H13, YTHDC1, and METTTL14 have been identified as potential markers for the diagnosis and prognosis of endometrial cancer. TIMER algorithm suggested that immune cell infiltration was related to the expression changes of ZC3H13, YTHDC1, and METTTL14. Meanwhile, ZC3H13 or YTHDC1 knockdown can promote the proliferation and invasion of endometrial cancer cells (96). Zhai et al. analyzed 406 cases of endometrial adenocarcinoma and 19 controls using a TCGA dataset. FTO, RBM15, and YTHDF1 were identified as independent prognostic markers for endometrial cancer, and FTO and RBM15 were differentially expressed between endometrial adenocarcinoma and hyperplasia. These data suggest that FTO, RBM15, and YTHDF1 are critical in the progression and prognosis of endometrial cancer (82, 97). Interestingly, Zhang et al. found that CpG sites located at the m^6^A regulatory site may be considered a potential prognostic feature of endometrial cancer patients (98). In a more detailed study, 19 m^6^A RNA methylation regulators were abnormally expressed in endometrial carcinoma. Univariate and multivariate Cox regression analyses showed that age, grade, and risk score were independent risk factors. High FTO expression was associated with poor overall survival (99).

### 3.3 m^6^A in Ovarian Cancer

#### 3.3.1 Function of m^6^A on mRNA in Ovarian Cancer

Ovarian cancer is one of the most deadly gynecological malignancies. It often leads to poor prognosis due to the insidious onset and lack of effective early detection indicators (100). YTHDF1 is a member of the YT521-B homologous domain (YTH) protein family. The protein recognizes m^6^A through a conserved aromatic cage in its YTH domain and mediates gene regulation at the post-transcriptional level (52). Hao et al. found that TRIM29 expression increased at the translational level in cisplatin-resistant ovarian cancer cells and clinical tissues. Increased TRIM29 expression is associated with poor prognosis in patients with ovarian cancer. In addition, YTHDF1’s recruitment of m^6^A-modified TRIM29 is involved in promoting TRIM29 translation in cisplatin-resistant ovarian cancer cells. Knockout of YTHDF1 inhibits the tumor stem cell-like characteristics of cisplatin-resistant ovarian cancer cells, which can be salvaged by ectopic expression of TRIM29 (101). YTHDF1, as an upstream molecule of TRIM29, can recognize its 3’UTR and promote its translation in ovarian cancer. Thus, TRIM29 is expected to be a potential therapeutic target for ovarian cancer. In addition, YTHDF1 can promote ovarian cancer progression by increasing EIF3C translation. YTHDF1, as a direct target, binds to m^6^A-modified EIF3C mRNA to increase EIF3C translation in an m^6^A-dependent manner and simultaneously promotes overall translation output. Thus, the occurrence and metastasis of ovarian cancer are promoted (102). Therefore, the YTHDF1-EIF3C axis is expected to be a target for the development of cancer treatment drugs.

Another study showed that the RNA demethylase ALKBH5 was upregulated in ovarian cancer tissues but lower in ovarian cancer cell lines than in normal ovarian cell lines. Interestingly, the molecular function toll-like receptor (TLR4) in the tumor microenvironment also showed the same expression trend. NANOG is a target of ALKBH5-mediated m6A modification. The expression of NANOG increased after mRNA demethylation, thereby enhancing the aggressiveness of ovarian cancer cells. In addition, the high expression of TLR4 activated the NF-Kappa B pathway, upregulated the expression of ALKBH5, and increased the level of m^6^A and the expression of NANOG, all of which contributed to the occurrence of ovarian cancer (103). FBW7, a tumor suppressor, is a substrate recognition component of the SCF e3-ubiquitin ligase complex, which mediates protein degradation of various carcinogenic proteins. Xu et al. used MeRIP-Seq and RNA-Seq to evaluate downstream targets of YTHDF2. They found that FBW7 was significantly down-regulated in ovarian cancer tissues, and its high expression was associated with a good prognosis and increased m^6^A modification. FBW7 counteracts the tumor-promoting effect of YTHDF2 by inducing proteasome degradation of YTHDF2 in ovarian cancer. In addition, YTHDF2 can regulate the turnover of m^6^A-modified mRNA, including pro-apoptotic gene BMF. Therefore, FBW7 inhibits tumor growth and progression by antagonizing the YTHDF2-mediated attenuation of BMF mRNA in ovarian cancer tissues (28). FTO, an m^6^A demethylase, plays an important role in the progression of ovarian cancer. One study found that FTO enhanced the second messenger 3’, 5’ -cyclic adenosine phosphate (cAMP) signaling by decreasing the m^6^A modification level of 3’ UTR and the mRNA stability of two phosphodiesterase genes (PDE1C and PDE4B), inhibiting the dry character of ovarian cancer cells. Therefore, FTO plays a tumor suppressor role in ovarian cancer by inhibiting cAMP signaling (104).

**Figure 4:**
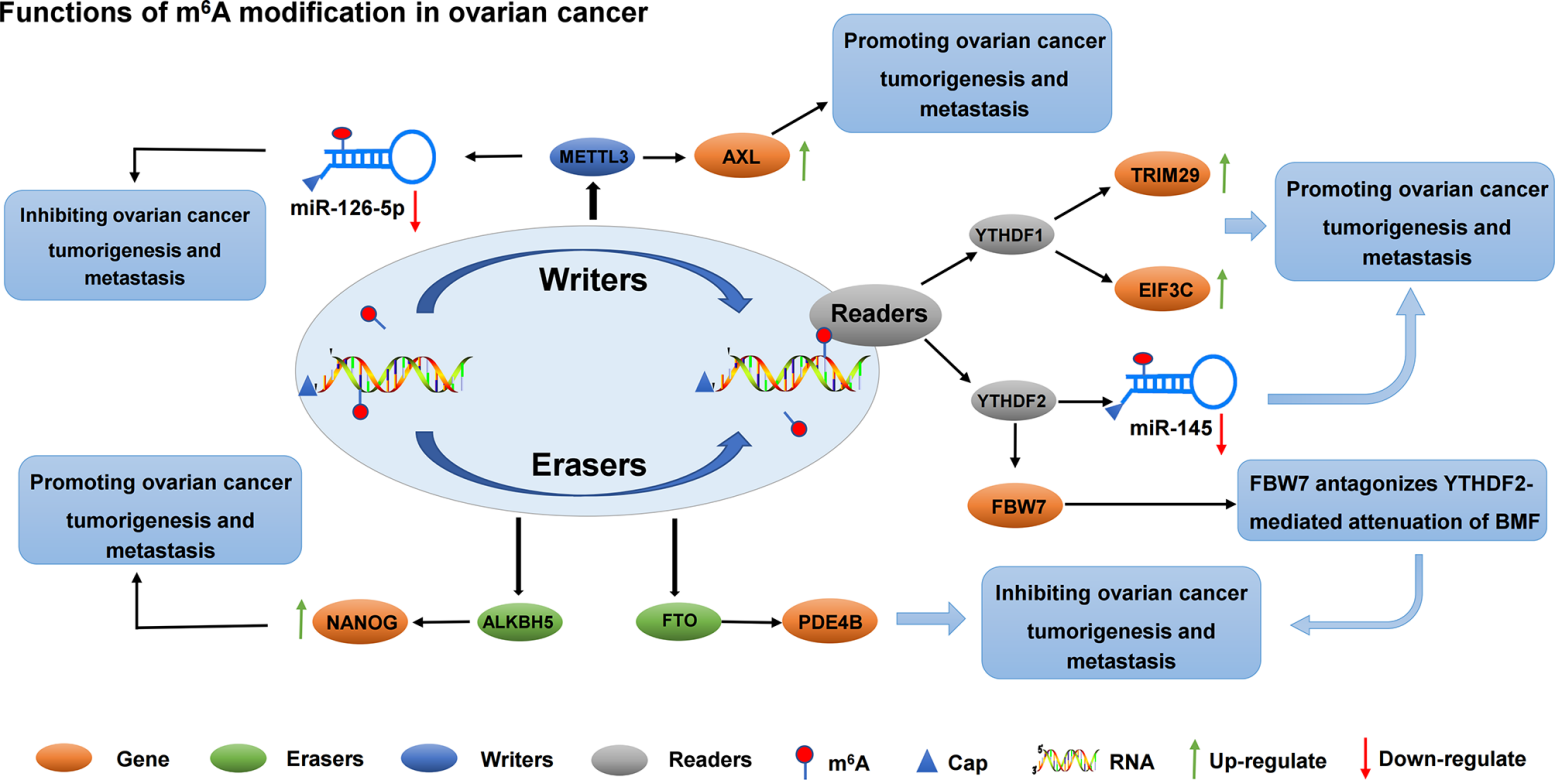
The roles of different m^6^A regulators in regulating RNAs in ovarian cancer. m^6^A methylation regulators METTL3, YTHDF1, YTHDF2, FTO and ALKBH5 promote/inhibit the invasion and metastasis of ovarian cancer by binding to mRNA and regulating mRNA expression. In addition, METTL3 regulates the expression of miR-126-5P and inhibits the proliferation and metastasis of ovarian cancer. YTHDF2 regulates the expression of miR-145 and promotes the proliferation and metastasis of ovarian cancer.

Progress has also been made in the role of methyltransferase in ovarian cancer. For example, methyltransferase METTL3 not only promotes the growth and invasion of ovarian cancer by regulating AXL translation and epithelial-to-mesenchymal transformation (105), but also plays a carcinogenic role in the progression of ovarian cancer cells by regulating the phosphorylation of AKT and the expression of Cyclin D1, a downstream effector (106). Thus, METTL3 may be a new prognostic and/or therapeutic target for ovarian cancer. The roles of different m^6^A regulators in regulating RNAs in ovarian cancer are shown in **Figure 4** and **Table 3**.

**Table 3:** The roles of different m^6^A regulators in regulating RNAs in ovarian cancer.

m^6^A regulators	Genes/RNAs	Cell lines	Location	Role	Mechanism	Function	References
YTHDF1	TRIM29	SKOV3, A2780, SKOV3/DDP, A2780/DDP	mRNA	Oncogene	Enhance expression of TRIM29	Promoting ovarian cancer tumorigenesis and metastasis	(101)
YTHDF1	EIF3C	HEK293T, A2780, SKOV3	mRNA	Oncogene	Enhance expression of EIF3C	Promoting ovarian cancer tumorigenesis and metastasis	(102)
ALKBH5	NANOG	SKOV3, HEY, HO8910, OVCAR3, Ishikawa	mRNA	Oncogene	Enhance expression of NANOG	Promoting ovarian cancer tumorigenesis and metastasis	(103)
YTHDF2	FBW7	SKOV3, OVCA420, OVCA429, OVCAR8	mRNA	Tumor suppressor	FBW7 antagonizes YTHDF2-mediated attenuation of BMF	Inhibiting ovarian cancer tumorigenesis and metastasis	(28)
FTO	PDE1C	SKOV3, HEK293T, COV362, OVCAR5	mRNA	Tumor suppressor	Decreased m^6^A modification level and phosphodiesterase gene stability	Inhibiting ovarian cancer tumorigenesis and metastasis	(104)
FTO	PDE4B	SKOV3, HEK293T, COV362, OVCAR5	mRNA	Tumor suppressor	Decreased m^6^A modification level and phosphodiesterase gene stability	Inhibiting ovarian cancer tumorigenesis and metastasis	(104)
METTL3	AXL	A2780, COV504, ES2, HO-8910, OVCAR3, SKOV3	mRNA	Oncogene	Regulates AXL translation and epithelial-to-mesenchymal transformation	Promoting ovarian cancer tumorigenesis and metastasis	(105, 106)
YTHDF2	miR-145	SKOV3, 3AO	miRNA	Oncogene	Down-regulated miR-145	Promoting ovarian cancer tumorigenesis and metastasis	(107)
METTTL3	miR-126-5p	A278, COV504, SKOV3, ES2, IOSE-80	miRNA	Tumor suppressor	Down-regulated miR-126-5p	Inhibiting ovarian cancer tumorigenesis and metastasis	(108)

#### 3.3.2 Function of m^6^A on ncRNA in Ovarian Cancer

RNA methylation can be methylated at the RNA level, which is an extremely important epigenetic modification. Methylation of RNA m^6^A was correlated with miRNA. On the one hand, the target sites of miRNA showed m^6^A enrichment, and miRNA positively regulated m^6^A modification activity. On the other hand, miRNA synthesis relies on m^6^A methylation modification (109). Li et al. found, by studying how YTHDF2 and miR-145 regulate the progression of ovarian cancer through m^6^A modification, that YTHDF2 was significantly up-regulated in ovarian cancer tissues compared with normal ovarian tissues. Meanwhile, YTHDF2 significantly promoted the proliferation and migration of ovarian cancer cell lines, and reduced the modification level of m^6^A mRNA. The expression level of miR-145 in ovarian cancer tissues and cells was negatively correlated with that of YTHDF2, which is the direct target gene of miR-145. Key crosstalk occurred between miR-145 and YTHDF2 through a double negative feedback loop. The overexpression of YTHDF2 rescues the decreased proliferation and migration of miR-145-induced ovarian cancer cells, suggesting a new target for the treatment of ovarian cancer (107). Another miRNA, miR-126-5p, is up-regulated in ovarian cancer. The overexpression of miR-126-5P can promote proliferation, migration, and invasion of ovarian cancer cells and inhibit apoptosis. In addition, miR-126-5p activates the PI3K/Akt/mTOR pathway by targeting PTEN. Moreover, RNA methyltransferase METTL3 promoted the maturation of miR-126-5p through m^6^A modification of pri-miR-126-5p. Finally, *in vitro* and *in vivo* experiments confirmed that METTL3 silencing blocks the PI3K/AKT/mTOR pathway by disrupting miR-126-5P targeted inhibition of PTEN, thereby hindering ovarian cancer progression and tumorgenesis. Therefore, the down-regulation of METTL3 can inhibit the up-regulation of PTEN by miR-126-5p, and prevent the activation of PI3K/AKT/mTOR pathway, inhibiting the occurrence and development of ovarian cancer (108). The role and possible mechanisms of circRNAs in autophagy in ovarian cancer have not been systematically studied. Zhang et al. screened circRNA, miRNA, and mRNA expression profiles of Torin 1-induced ovarian cancer cells. They found that circRNA circRAB11FIP1 was up-regulated in ovarian cancer cells, and silencing circRAB11FIP1 could inhibit autophagy of ovarian cancer cells. CIRCRAB11FIP1-induced autophagy accelerated the proliferation and invasion of ovarian cancer cells. In addition, circRAB11FIP1 directly binds to Mir-129 and regulates its targets ATG7 and ATG14. CircRAB11FIP1 mediates ATG5 and ATG7 mRNA expression levels depending on m^6^A modification (110). Few studies on lncRNAs in ovarian cancer have also been conducted. In recent years, studies have found that lncRNA is an important functional regulator in ovarian cancer. Wang et al. found that lncRNA RHPT1-AS1 was up-regulated in ovarian cancer tissues and was closely associated with poor prognosis. However, m^6^A modification improved the stability of RHPT1-AS1 methylated transcription by reducing RNA degradation, leading to the up-regulation of RHPT1-AS1 expression in ovarian cancer and promoting the proliferation and metastasis of ovarian cancer (111).

#### 3.3.3 Prognostic Effect of m^6^A RNA Methylation Regulators on Ovarian Cancer

m^6^A RNA methylation is involved in the initiation and progression of various cancers. Therefore, m^6^A RNA methylation regulators are greatly important in tumor prognosis. Fan et al. analyzed the prognostic value of the transcription levels of 18 m^6^A RNA methylation regulators in ovarian cancer and found that IGF2BP1, VIRMA, and ZC3H13 predicted the highest prognostic score of ovarian cancer. Therefore, the authors suggest that IGF2BP1, VIRMA, and ZC3H13 mRNA levels are important factors in predicting prognosis and developing treatment strategies (112). In addition, Han et al. downloaded the mutation and copy number variation (CNV) data from 579 ovarian cancer patients from TCGA database and analyzed gene expression and prognostic value using integrated bioinformatics. Bioinformatics and Cox multivariate analysis showed the significant correlation between high expression of WTAP and ovarian cancer prognosis (113). Meanwhile, analysis of other gene sets found that the prognosis of ovarian cancer was associated with HNRNPA2B1, KIAA1429, and WTAP (114). Na et al. identified NEBL, PDGFRA, WDR91, and ZBTB4 genes as potential independent prognostic risk characteristics of ovarian cancer (115).

## 4 Conclusions and Perspectives

RNA m^6^A modification, as a hotspot of epigenetics research, plays a crucial role in the post-transcriptional regulation of gene expression; it has attracted increased attention. It is also involved in various biological processes and disease progression. RNA m^6^A modification plays an important role in promoting or inhibiting the growth, proliferation, migration, invasion, specific metastasis, drug resistance, and prognosis of gynecological cancers through three effector factors, namely, writer, erasers, and readers. From the viewpoint of the epitome, the tissue specificity and uneven distribution of m^6^A modification provide new directions for understanding the pathogenesis of multiple diseases, especially tumors. m^6^A modification is a “double-edged sword”, promoting or inhibiting the occurrence and development of tumor mainly by regulating the mRNA level of oncogene or tumor suppressor gene. The role of m^6^A modification in gynecological cancer is further clarified with the deepening of the research on the network mechanism of m^6^A modification regulation.

At present, studies on the biological effects of m^6^A modification on gynecological cancers have made some progress, but some problems should still be further studied and solved. Multiple studies have shown that m^6^A RNA methylation regulators have the potential for prognostic assessment and as biomarkers for early diagnosis of gynecological cancers. For example, METTL3, ZC3H13, YTHDC1, and YTHDF1 have been found to be prognostic markers of cervical cancer (84). IGF2BP3, KIAA1429, IGF2BP1, YTHDF3, ZC3H13, YTHDC1, and METTL14 can be used as prognostic markers of endometrial cancer (93, 94). HNRNPA2B1, KIAA1429, and WTAP can be used as prognostic markers of ovarian cancer (114). However, these studies are based only on systematic analyses of public databases. The prognostic ability of m^6^A RNA methylation regulators in gynecological cancers remains limited due to the difficulty of obtaining sufficient detection samples. Therefore, large-scale experimental verification and clinical trials on m^6^A modification should be conducted to remedy this defect in future studies. In addition, most current studies on m^6^A modification in gynecological cancer are limited to the mechanism of action in gynecological cancer cells. Thus, more translational studies are required in the future to further clarify the use of m^6^A alone or in combination with other therapies for gynecological cancers for effective application to clinical treatment. Finally, the immune system is the host’s defense system against infection and disease. Meanwhile, immunotherapy is a new cancer treatment strategy, which has been widely used to treat various solid tumors, including gastrointestinal tumors and gynecological tumors (116, 117). In recent years, m^6^A regulatory factors have been widely studied in tumor immunotherapy and immune evasion. Moreover, tumor immunotherapy is the most promising therapeutic strategy, and m^6^A modification-mediated immune invasion becomes a hotspot in the study of the pathogenesis and prognosis of gynecological tumors. However, the study of immune invasion mediated by m^6^A modification in gynecological tumors is still in its infancy, and m^6^A modification is expected to make new breakthroughs in this field in future studies.

## Author Contributions

WH, FK, and KW contributed to conceive and design the study. RL and XC performed the systematic searching. WH and RL extracted the data. FK and KW wrote the manuscript. XC and WH supervised the manuscript. All of the authors read and approved the final manuscript.

## Conflict of Interest

The authors declare that the research was conducted in the absence of any commercial or financial relationships that could be construed as a potential conflict of interest.

## Publisher’s Note

All claims expressed in this article are solely those of the authors and do not necessarily represent those of their affiliated organizations, or those of the publisher, the editors and the reviewers. Any product that may be evaluated in this article, or claim that may be made by its manufacturer, is not guaranteed or endorsed by the publisher.
